# Optogenetic stimulation of medial prefrontal cortex *Drd1* neurons produces rapid and long-lasting antidepressant effects

**DOI:** 10.1038/s41467-018-08168-9

**Published:** 2019-01-15

**Authors:** Brendan D. Hare, Ryota Shinohara, Rong Jian Liu, Santosh Pothula, Ralph J. DiLeone, Ronald S. Duman

**Affiliations:** Department of Psychiatry, Yale University School of Medicine, New Haven, CT 06519 USA

## Abstract

Impaired function in the medial prefrontal cortex (mPFC) contributes to depression, and the therapeutic response produced by novel rapid-acting antidepressants such as ketamine are mediated by mPFC activity. The mPFC contains multiple types of pyramidal cells, but it is unclear whether a particular subtype mediates the rapid antidepressant actions of ketamine. Here we tested two major subtypes, *Drd1* and *Drd2* dopamine receptor expressing pyramidal neurons and found that activating *Drd1* expressing pyramidal cells in the mPFC produces rapid and long-lasting antidepressant and anxiolytic responses. In contrast, photostimulation of *Drd2* expressing pyramidal cells was ineffective across anxiety-like and depression-like measures. Disruption of *Drd1* activity also blocked the rapid antidepressant effects of ketamine. Finally, we demonstrate that stimulation of mPFC *Drd1* terminals in the BLA recapitulates the antidepressant effects of somatic stimulation. These findings aid in understanding the cellular target neurons in the mPFC and the downstream circuitry involved in rapid antidepressant responses.

## Abstract (web summary)

Ketamine exerts fast-acting anti-depressant responses. Here the authors show that dopamine D1 receptor expressing neurons in the medial prefrontal cortex contribute to these antidepressant-like effects in mice.

## Introduction

Ketamine’s rapid antidepressant response^1^, and its efficacy in treatment-resistant individuals^2^, is arguably the most significant advance in the treatment of mood disorders in over 60 years. Preclinical studies indicate that glutamatergic signaling in the medial prefrontal cortex (mPFC) is critical to the therapeutic actions of ketamine, an *N*-methyl-d-aspartate (NMDA) antagonist. A low, sub-anesthetic dose of ketamine produces a paradoxical burst of glutamate in the mPFC^3^, and neuronal silencing of the mPFC blocks the antidepressant actions of ketamine^4^. A key role for mPFC is also supported by optogenetic studies demonstrating that photostimulation of *Camk2a* expressing mPFC pyramidal neurons is sufficient to reproduce the rapid and sustained antidepressant behavioral actions of ketamine^4^.

The mPFC serves as a central hub that can shape the activity in a distributed network of output structures, including regulation of behavioral and autonomic responses to stress. While there have been efforts to characterize the role of interneuron subtypes in stress and depression models^5,6^, less is known about subtypes of pyramidal neurons. Different types of pyramidal neurons have been described based on morphological and electrophysiological properties, as well as projection targets^7^ but the actions of these subtypes in models of depression are unclear. Additionally, while the burst of glutamatergic signaling in the mPFC following ketamine may impact subtypes indiscriminately, the antidepressant response driven by this activation may be subtype specific.

Two major subtypes of principal neurons referred to as type A and type B have been characterized in the mPFC. Type A neurons are found predominantly in deep layers while type B neurons are distributed through deep and superficial layers of the mPFC, and type A and B neuron projections are largely non-overlapping^8,9^. Type A neurons have a more complex dendritic arborization than type B neurons, and have a prominent voltage sag in response to current injection^8,9^. The mPFC receives afferent input from multiple regions. In many cases, how these inputs target type A and type B cells is unclear, though callosal and hippocampal inputs appear to elicit responses in both types^10^ potentially in a subregion-specific fashion^11^. *Drd1* and *Drd2* dopamine receptor gene expression is segregated in type B and type A cells, respectively^12^, and *Drd1*-positive neurons exhibit the sparse dendritic morphology typical of type B cells^13^. Consequently, type B cells can be targeted by expressing cre-recombinase (Cre) under control of the *Drd1* promoter, while the type A population can be targeted using Cre expression under control of *Drd2*^9^.

The roles of *Drd1*/type B and *Drd2*/type A cells in antidepressant responses have not been tested. However, repeated stress paradigms, which are used for preclinical studies of depression, attenuate working memory via a reduction in the activity of *Drd1* expressing pyramidal cells in mPFC^14^. In addition, repeated stress exposure attenuates dopamine D1 receptor (D1r) signaling and causes atrophy of *Drd1*-expressing neurons^15^. These findings suggest that stress-sensitive circuits involving mPFC *Drd1-*expressing neurons may represent therapeutic targets of ketamine. Here we test this hypothesis by selectively manipulating *Drd1*/type B vs. *Drd2/*type A cell activity using Cre-inducible transgenic lines to determine if one or both of these principal neuron subtypes mediate the rapid antidepressant actions of ketamine. The results demonstrate that prior stimulation of *Drd1*, but not *Drd2-*expressing pyramidal neurons, in the mPFC produces rapid and sustained antidepressant responses, and that activation of *Drd1* neurons is required for the antidepressant actions of ketamine.

## Results

### Characterization of *Drd1* and *Drd2* neurons in the mPFC

**Fig. 1:**
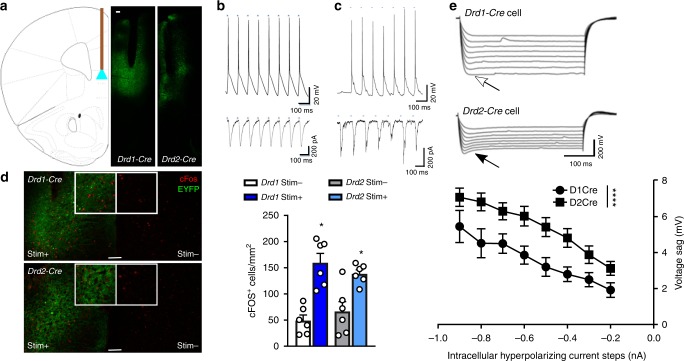
Cre-dependent targeting of Chr2 to pyramidal cell subtypes in the mouse mPFC. **a** mPFC targeting strategy and representative images of Chr2 expression and cannula placement in *Drd1-Cre* and *Drd2-Cre* mice. **b**, **c** Action potential generation and inward current produced by light activation in *Drd1-Cre* and *Drd2-Cre* pyramidal cells, respectively. **d** Unilateral stimulation of Chr2 in *Drd1-Cre* and *Drd2-Cre* mice produces similar increases in cFos expression (normalized cells per mm^2^) in the stimulated hemisphere that are greater than those observed in the unstimulated hemisphere (Wilcoxon matched pairs signed rank, *p < 0.05 vs. respective stim−, *n* = 6 males/group). **e** Light-sensitive pyramidal cells in *Drd1-Cre* mice demonstrate lower voltage sag (open arrow) upon current injection than light-sensitive pyramidal cells in *Drd2-Cre* mice (closed arrow) (Factorial ANOVA, group ****p < 0.0001, *Drd1-Cre* 9 cells/2 male animals, *Drd2-Cre* 10 cells/3 male animals). Error bars represent mean ± SEM, scale bar 100 µM

A Cre-dependent channel rhodopsin viral vector (AAV-Chr2) was used to selectively express Chr2 in the mPFC of *Drd1-* and *Drd2*-*Cre recombinase* mice, resulting in Chr2-EYFP fluorescence in the targeted area. Fiber optic cannula tips were positioned to direct light into the ventral portion of mPFC (Fig. 1a) based on previous work^4^. Patch clamp recordings of mPFC slices from *Drd1*-Chr2-EYFP (Fig. 1b) and *Drd2*-Chr2-EYFP (Fig. 1c) cells in layer V of the mPFC demonstrate high fidelity action potential production and inward current in response to laser stimulation (15 ms, 10 hz, 473 nm). Neuronal activation in vivo was examined by cFos labeling after unilateral infusion of AAV-Chr2 and optic fiber implant into the mPFC of *Drd1*-Cre and *Drd2-*Cre mice. Optogenetic stimulation (10 hz, 15 ms, 1 min-on/1 min-off, 20 min) of *Drd1-Cre* and *Drd2-Cre* mice produced a robust increase in cFos-positive neurons in comparison to labeling in the unstimulated, contralateral side (Fig. 1d) and non-Chr2-expressing EYFP controls (*p* < 0.001, comparison in Supplementary Table 1). These findings demonstrate that photostimulation of pyramidal cells in *Drd1-* or *Drd2-Cre mice* produces similar action potentials and immediate early gene expression consistent with neuronal activation of *Drd1* and *Drd2* neurons in both lines of mice.

We next tested for the presence or absence of a prominent voltage sag in response to hyperpolarizing current that is characteristic of these pyramidal neuron subtypes. Consistent with previous reports demonstrating that approximately 74% of *Drd2* cells exhibit prominent voltage sag, but only 4% of *Drd1* cells have a similar response^9,16^, blue light-sensitive cells in *Drd2-Cre* mice displayed a larger voltage sag than photosensitive cells in *Drd1-Cre* mice (Fig. 1e). As expected, both lines also had unlabeled, photo-insensitive cells that displayed the alternate phenotype (i.e., *Drd1*-Cre mice had photo-insensitive cells with high voltage sag, and *Drd2-*Cre mice, photo-insensitive cells with low voltage sag; Supplementary Fig. 1A–D). These results demonstrate that Cre-dependent Chr2 expression in *Drd1-Cre* and *Drd2-Cre* mice can be utilized to target separate populations of pyramidal cells.

We next conducted studies to determine if ketamine selectively blocks NMDA receptors on type A and type B cells (Supplementary Fig. 2A), identified by the magnitude of voltage sag as demonstrated in Fig. 1e. NMDA application produced a greater increase in inward current in type A cells as compared to type B cells (Supplementary Fig. 2B), but ketamine at concentrations of 1 and 10 µM produced a similar blockade of NMDA receptor currents in both cell types (Supplementary Fig. 2C). However, it remains unclear whether the glutamate burst that occurs following ketamine administration produces antidepressant effects via actions on one or the other cell type. To address this question, we utilized *Drd1-* and *Drd2-Cre* mice to selectively manipulate type B and type A cells in vivo, respectively, and examined the antidepressant behavioral responses.

### *Drd1-* and *Drd2-Cre* mice display similar behaviors

To test for behavioral changes that might result from *Cre*-transgene expression, we conducted a series of behavioral tests to compare *Drd1-Cre* and *Drd2-Cre* mice with wild-type littermate controls that lack the *Cre*-transgene (*Drd1-WT, Drd2-WT*). We first examined locomotor activity in animals individually exposed to a clean home cage. We found no difference in locomotion between *Cre* and *WT* animals of either genotype (Supplementary Fig. 3A, E). Similarly, along the same timeline to be used for the photostimulation studies of the forced swim test (FST, 6 min pre-swim, and 6 min test swim separated by 48 h), we found no significant differences between *WT* and *Cre* animals for either genotype (Supplementary Fig. 3B, F). Additionally, there were no effects on anxiety-like behavior on the elevated plus maze (EPM; Supplementary Fig. 3C, G), or on EPM arm entries (Supplementary Fig. 3D, H). Given the absence of a Cre-associated endophenotype, we compared *Cre* animals to *WT* littermate controls in the following experiments.

### *Drd1* stimulation drives antidepressant responses

To determine whether photostimulation of *Drd1*-expressing cells produces ketamine-like behavioral responses, *Drd1-Cre* mice were bilaterally infused with Cre-dependent AAV-Chr2 into the mPFC. *Drd1-WT* animals underwent similar stereotaxic surgery to infuse AAV-EYFP. Bilateral fiber optic cannula were placed 0.2 mm above the injection sites and behavioral testing began 14 days after the surgical procedure. Twenty-four hours after an initial swim exposure, the animals were tethered to a fiber optic rotary joint and photostimulated (5 mW/side, 10 hz, 15 ms, 1 min on/1 min off, 60 min total) in their home cage. These parameters generate high spike fidelity over long periods of stimulation^17,18^, and were used in prior studies of mPFC pyramidal cell activation^4^. Sixty minutes of stimulation was chosen to match the period of increased extracellular glutamate following sub-anesthetic ketamine administration^3^. Note that behavioral testing was not conducted during photostimulation, but started 24 h later and proceeded, in subsequent experiments, for up to 7 days to model the time course for the rapid and sustained antidepressant actions of ketamine^19^. Mice were subjected to a test swim 24 h after photostimulation, and 24 h after the test swim, a 10 min EPM exposure was conducted.

**Fig. 2:**
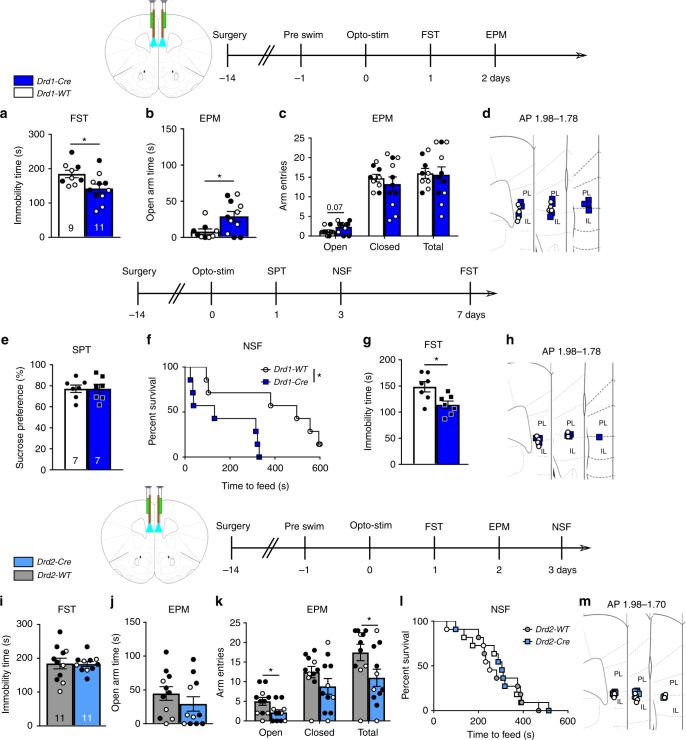
Antidepressant effect of prior stimulation of mPFC *Drd1* expressing cells. **a** *Drd1-Cre* forced swim immobility 24 h after photostimulation (unpaired *t*-test, *p* < 0.05). **b**, **c** *Drd1-Cre* elevated plus maze open arm exploration time and arm entries 48 h after photostimulation (unpaired *t*-test, *p* < 0.05). **d** Fiber optic cannula placements for photostimulation of *Drd1-Cre* mice. **e** *Drd1-Cre* sucrose preference outcome 24 h after photostimulation. **f** *Drd1-Cre* novelty suppressed feeding times 72 h after photostimulation (Mantel–Cox (log rank), *p* < 0.05). **g** *Drd1-Cre* total immobility time in the forced swim test 7 days after photostimulation (unpaired *t*-test, *p* < 0.05). **h** Second cohort *Drd1-Cre* fiber optic cannula placements. **i** *Drd2-Cre* forced swim immobility time 24 h after photostimulation. **j**, **k** *Drd2-Cre* elevated plus maze open arm time and arm entries 48 h after photostimulation (unpaired *t*-test, *p* < 0.05). **l** *Drd2-Cre* time to feed in the novelty suppressed feeding test 72 h after photostimulation. **m** Fiber optic cannula locations for photostimulation of *Drd2-Cre* mice; *n* = **a**–**d** *Drd1-Cre* 5 males, 6 females; WT 4 males, 5 females, **e**–**h** *Drd1-Cre* 7 males, WT 7 males, **i**–**m** *Drd2-Cre* 7 males, 4 females; WT 7 males, 4 females; *p < 0.05. Error bars represent mean ± SEM. Female—closed circles, male—open circles

Photostimulation of the mPFC in *Drd1-Cre* mice produced an antidepressant response in the FST, measured by decreased time immobile compared to *Drd1*-*WT* control mice (Fig. 2a). This demonstrates that photostimulation results in adaptive plasticity produced by the mPFC *Drd1*-expressing pyramidal cell population that is capable of supporting an antidepressant response within 24 h after light delivery. In the EPM conducted 48 h after photostimulation, *Drd1-Cre* mice spent more time in the open arms and displayed a trend for increased open arm entries, indicating reduced anxiety (Fig. 2b); there were no changes in closed arm entries or total arm entries (Fig. 2c).

Previous studies demonstrate that non-selective photostimulation of principal neurons (*Camk2a* positive) in the mPFC produces antidepressant actions in the sucrose preference and novelty suppressed feeding (NSF) tests^4^. In a second cohort of mice, we examined whether photostimulation of *Drd1-Cre* mice influences these behaviors and examined the duration of the FST effect. Unlike the previous studies in rats^4^, photostimulation 24 h prior to testing did not increase sucrose preference in *Drd1-Cre* mice (Fig. 2e). However, there was a clear effect of photostimulation to reduce latency to feed in the NSF test, another indication of reduced anxiety, measured 3 days after photostimulation (Fig. 2f). Home cage food consumption after the test demonstrated no difference between groups (Supplementary Fig. 7A). Finally, 7 days after photostimulation, an antidepressant response in the FST (i.e., decreased total immobility time) was observed in the *Drd1-Cre* mice (Fig. 2g). Taken together, the results demonstrate that photostimulation of *Drd1*-expressing pyramidal cells in the mPFC produces rapid and long-lasting antidepressant actions similar to ketamine.

Increased locomotion produced by photostimulation is a possible confound for these behaviors as increased activity could produce antidepressant- and anxiolytic-like effects, particularly in the FST (i.e., decreased immobility). In a separate cohort of mice, we found that photostimulation produced a real-time increase in locomotion in *Drd1-Cre* mice with a corresponding on/off time scale (Supplementary Fig. 4A). However, 24 h after the same photostimulation protocol used for the antidepressant behavioral studies, there was no increase, but rather a decrease in locomotor activity (Supplementary Fig. 4B). As such, locomotor confounds are unlikely to have contributed to the antidepressant and anxiolytic effects observed following photostimulation.

We next examined *Drd2-Cre* mice using the same photostimulation and behavioral testing timeline as in the initial *Drd1-Cre* experiment. Photostimulation of mPFC in *Drd2-Cre* mice had no effect on immobility time in the FST (Fig. 2i), or open arm time in the EPM, but significantly decreased open arm entries and total arm entries in the EPM (Fig. 2j, k). To further explore this subtle anxiety-like effect of photostimulation, we also conducted a NSF test in these mice, but found no difference in latency to feed at the 3 day time point (Fig. 2l); no effect on home cage food consumption (Supplementary Fig. 7B). These findings indicate that prior stimulation of the *Drd2*-expressing pyramidal cell population is not sufficient to produce antidepressant or anxiolytic responses.

### *Drd1* stimulation is required for ketamine responses

**Fig. 3:**
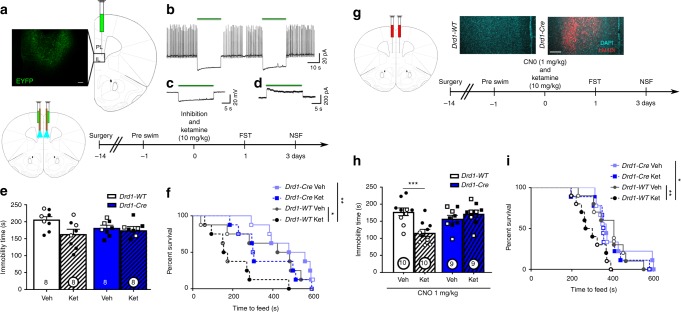
Inhibition of mPFC *Drd1* expressing cells blocks the antidepressant effect of ketamine. **a** Representative AAV-ARCH viral expression in ventral mPFC. **b** Action potentials induced by current injection in voltage clamp are potently inhibited by ARCH activation. **c** Current clamp recordings demonstrate hyperpolarization associated with light application. **d** Voltage clamp recordings demonstrate outward current during light application. **e** FST immobility time 24 h after photoinhibition and ketamine (Factorial ANOVA interaction *p* = 0.11). **f** Time to feed in the NSF test 72 h after photoinhibition and ketamine administration (Mantel–Cox (log rank) overall *p* < 0.01, WT-veh vs. WT-ket *p* < 0.05). **g** Representative AAV-hM4Di expression. **h** FST immobility time 24 h after hM4Di inhibition and ketamine (Factorial ANOVA interaction *p* < 0.01, post-hoc *t*-test *p* < 0.001). **i** Time to feed in the NSF 72 h after hM4Di inhibition and ketamine (Mantel–Cox (log rank) overall *p* < 0.05, WT-veh vs. WT-ket *p* < 0.01). **e**, **f** *n* = 5 males, 3 females per group; **h**, **i** *n* = 5 males, 5 females per WT group, *n* = 4 males, 5 females Cre ket, *n* = 5 males, 4 females Cre veh; *p < 0.05, **p < 0.01, ***p < 0.001. Error bars represent mean ± SEM, scale bar 100 µM. Male—closed data points, female—open data points

Based on these findings, we conducted studies to determine if the antidepressant actions of ketamine require activation of *Drd1*-expressing pyramidal cells using a photoinhibition approach. Viral *Cre*-dependent archaerhodopsin (AAV-eARCH3.0, referred to as ARCH, Fig. 3a, AAV-EYFP in WT animals) was infused into the mPFC of *Drd1-Cre* mice at the same coordinates as AAV-Chr2 in photostimulation experiments (Fig. 3a). In mPFC slices from these mice, spiking induced by somatic current injection in *Drd1* cells was potently inhibited by constant 561 nm light application (Fig. 3b). Similarly, light application induced hyperpolarization in current clamp (Fig. 3c), and outward current in voltage clamp (Fig. 3d) further demonstrating the functional inhibition of *Drd1* cells by ARCH. To further assess ARCH function in vivo, we examined the effects of photoinhibition on real-time locomotor behavior. In contrast to the clear changes in locomotor activation after stimulation of *Drd1* cells (Supplementary Fig. 4A), there was no reduction of activity during 1-min photoinhibition periods (Supplementary Fig. 4C). As this could result from a floor effect through habituation to the novel cage, a second cohort received continuous photoinhibition immediately upon entry into a novel cage (Supplementary Fig. 4D). This paradigm produced a reduction in activity in *Drd1-Cre* mice demonstrating a real time in vivo effect of photoinhibition that is opposite to photostimulation in *Drd1-Cre* mice.

Next, we tested the influence of photoinhibition of *Drd1* cells on the response to ketamine administration. Optogenetic inhibition (5 mW/side, 561 nm, constant light) of mPFC in *Drd1-Cre* mice was initiated concurrently with ketamine (10 mg/kg, i.p.) and continued for 1 h, the time period during which glutamate release is increased following ketamine administration^3^. Analysis of the FST results did not produce a significant interaction (genotype × treatment *p* = 0.11, Fig. 3e), likely due to the non-significant decrease in immobility time across the *Drd1-Cre* genotype. However, blockade of the ketamine response was confirmed in the NSF test. Ketamine administration significantly decreased the latency to feed in *Drd1-WT* mice compared to vehicle, and this effect was absent in *Drd1-Cre* mice (Fig. 3f). As in prior tests, this reduction in time to feed was not associated with changes in home cage feeding (Supplementary Fig. 7C).

Because the FST results were not clear in the photoinhibition study, we also utilized a chemogenetic inhibition approach, which provides a more sustained inhibition over the time period of ketamine exposure^20^. *Drd1* cells in the mPFC were targeted with a *Cre*-dependent inhibitory DREADD (AAV-hM4Di; Fig. 3g) 2 weeks prior to behavioral experiments. *Drd1*-*WT* and *Drd1*-*Cre* mice were treated with CNO (1 mg/kg) 30 min prior to ketamine or vehicle injection and tested 1 and 3 days later. Again, ketamine produced a reduction in FST immobility (Fig. 3h) and time to feed in the NSF test (Fig. 3i) in *Drd1-WT* mice not expressing hM4Di, and these effects in both the FST and NSF test were completely blocked in *Drd1-Cre* mice expressing the inhibitory DREADD (home cage food consumption Supplementary Fig. 7D). Ketamine induction of cFos-positive cells was significantly blocked in *Drd1-Cre* mice, confirming that photoinhibition produced a functional cellular response. There was a significant genotype and treatment effect, with cFos levels in ketamine-treated *Drd1-Cre* mice equivalent to those in vehicle-treated *Drd1-WT* mice (Supplementary Fig. 5). Together, these results demonstrate that activity in *Drd1* pyramidal cells is necessary for the antidepressant actions of ketamine.

### *Drd2* stimulation is not required for ketamine responses

**Fig. 4:**
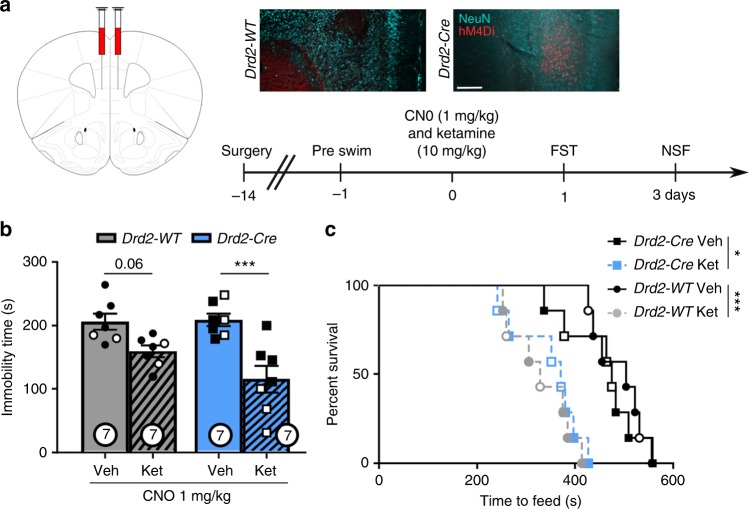
mPFC *Drd2-*expressing cells are not necessary for the ketamine response. **a** Representative AAV-hM4Di viral expression in ventral mPFC. **b** Forced swim immobility time 24 h after hM4Di inhibition and ketamine (post-hoc *t*-test, *p* < 0.001). **c** Time to feed in novelty suppressed feeding test 72 h after photoinhibition and ketamine administration (Mantel–Cox (log rank) overall *p* < 0.0001, WT-veh vs. WT-ket *p* < 0.0001, Cre-veh vs. Cre-ket *p* < 0.05). *n* = 5 males, 2 females per WT group, *n* = 4 males, 3 females per Cre group; *p < 0.05, ***p < 0.001. Error bars represent mean ± SEM, scale bar 100 µM. Male—closed data points, female—open data points

Photostimulation of mPFC *Drd2* cells was not sufficient to produce antidepressant or anxiolytic responses, but it is possible that plasticity of these cells is required for the sustained actions of ketamine. To test this hypothesis, we used the same DREADD-chemogenetic inhibition approach implemented in *Drd1* cells. *Drd2* cells in the mPFC were targeted with a Cre-dependent inhibitory DREADD (AAV-hM4Di; Fig. 4a) and *Drd2*-*WT* and *Drd2*-*Cre* mice were treated with CNO (1 mg/kg) 30 min prior to ketamine or vehicle injection. Contrary to our findings in *Drd1-Cre* mice, ketamine produced a clear antidepressant effect in the FST in both the *Drd2-WT* and *Drd2-Cre* mice expressing the inhibitory DREADD (Fig. 4b). In addition, ketamine produce an anxiolytic response in the NSF test in both the *Drd2-WT* and *Drd2-Cre* mice (Fig. 4c; home cage food consumption Supplementary Fig. 7E). Together, these results demonstrate that the mPFC *Drd2* cells are not required for the antidepressant response to ketamine.

### *Drd1-*BLA terminal stimulation drives antidepressant responses

To examine the potential projection regions that underlie mPFC *Drd1* cell photostimulation, we analyzed cFos immunolabeling in different brain regions. We observed that photostimulation of mPFC in *Drd1-Cre*, but not *Drd2*, mice significantly increased cFos in the basolateral amygdala (BLA) and anterior-dorsal bed nucleus of the stria terminalis (BNST) (Supplementary Fig. 6). We did not observe an increase in cFos in the dorsal raphe nucleus (DRN) after *Drd1* or *Drd2* cell stimulation, although previous studies report a role for mPFC to DRN projections in antidepressant behaviors^21,22^. The BLA is an important component of the stress reactivity and fear learning circuitry that modulates behavioral responses to stimuli of positive or negative valence^23^. A previous study of feeding behavior reported that photostimulation of mPFC *Drd1* cells increased cFos in the BLA, primarily in principal neurons^24^. BLA projection neurons have reciprocal connections with the mPFC, as well as projections to the ventral hippocampus, nucleus accumbens, and BNST^25,26^. Together these studies are consistent with the possibility that the BLA mediates the antidepressant actions of mPFC photostimulation.

**Fig. 5:**
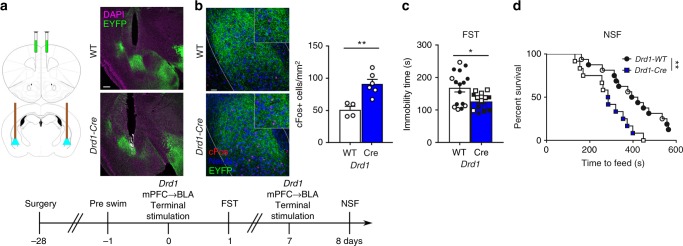
Antidepressant effect of prior stimulation of mPFC *Drd1* terminals in the BLA. **a** Experimental strategy (infusions and cannulations were bilateral) and representative cannula placements over mPFC *Drd1-Cre* or *WT* BLA terminals (green—EYFP, magenta—DAPI); scale bar 100 µM. **b** Photostimulation of mPFC *Drd1* cell terminal field in the BLA increases cFos expression (normalized cells per mm^2^, Mann–Whitney, *p* < 0.01, *Drd1-Cre* (*n* = 5) vs. *Drd1-WT* (*n* = 4), green—EYFP, red—cFos, blue—NeuN). **c** FST immobility time 24 h after photostimulation of mPFC *Drd1* terminals in the BLA (Mann–Whitney *p* < 0.05) *Drd1-WT*, 11 males, 5 females; *Drd1-Cre*, 7 males, 7 females. **d** NSF time 24 h after photostimulation of mPFC *Drd1* terminals in the BLA (Mantel–Cox (log rank) *p* < 0.01). *Drd1-WT*, 11 males, 5 females; *Drd1-Cre*, 6 males, 6 females; *p < 0.05, **p < 0.01. Error bars represent mean ± SEM. Male—closed data points, female—open data points

To examine the role of specific mPFC *Drd1* projections in the rapid antidepressant response produced by somatic stimulation, we photostimulated mPFC *Drd1* terminals within the BLA. AAV-Chr2 was infused into the mPFC of *Drd1-Cre* mice and a fiber optic was placed over the BLA (Fig. 5a). Photostimulation of mPFC terminals in the BLA produced a robust cFos signal (Fig. 5b) compared to EYFP-expressing WT mice. The behavioral effects of photostimulation of mPFC *Drd1* cell terminals in the BLA were examined in WT and *Drd1-Cre* mice using the same stimulation protocol (5 mW/side, 10 hz, 15 ms, 1 min on/1 min off, 60 min total) as for somatic stimulation. Photostimulation of BLA terminals produced an antidepressant response in the FST at 24 h after stimulation (Fig. 5c). Preliminary studies conducted 72 h after photostimulation showed no effect in the NSF test, possibly due to reduced impact of terminal stimulation compared to somatic stimulation. Therefore, mice received a second terminal stimulation (7 days after the first) and when tested 24 h later, showed an anxiolytic response in the NSF test (Fig. 5d, home cage food consumption Supplementary Fig. 7F). These findings highlight the BLA as critical site associated with the rapid and prolonged antidepressant response to mPFC *Drd1* terminal stimulation.

**Fig. 6:**
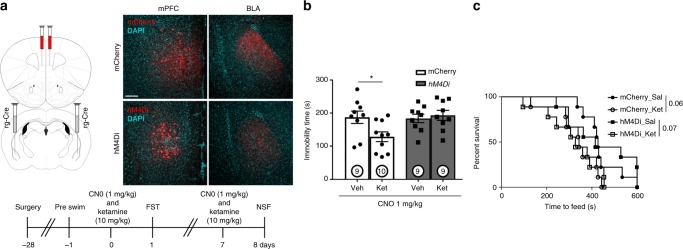
mPFC projections to the BLA play a role in the ketamine response. **a** Viral strategy and representative mCherry and hM4Di expression (red = mCherry, hM4D1, cyan = DAPI). **b** FST immobility time 24 h after CNO and ketamine administration (factoral ANOVA interaction *p* < 0.05, post-hoc *t*-test *p < 0.05). **c** Time to feed in the NSF test 24 h after CNO and ketamine administration (Mantel–Cox (log rank) overall *p* = 0.06); *n* = 9 mCherry Veh, hM4Di Veh, hM4Di Ket, *n* = 10 mCherry Ket. All male. Error bars represent mean ± SEM, scale bar 100 µM

Next, we tested the role of mPFC-BLA circuitry in the response to ketamine by selectively inhibiting projections to the BLA. Retrograde-Cre (AAVrg-Cre) viral infusions targeting the BLA were paired with Cre-dependent inhibitory DREADD hM4Di or mCherry control virus infusions into the mPFC to selectively inhibit mPFC projections to the amygdala during ketamine administration (Fig. 6a). Mice received CNO (1 mg/kg) 30 min prior to ketamine or vehicle injection and 24 h later were tested in the FST. Ketamine administration significantly decreased FST immobility in the control mCherry mice, and this antidepressant response was completely blocked in mice infused with the Cre-dependent inhibitory DREADD (Fig. 6b). However, 24 h after a second treatment, mPFC to BLA inhibition failed to block a trend-level reduction in feeding time in the NSF test (Fig. 6c, home cage food consumption Supplementary Fig. 7G). These findings indicate that mPFC projections to the BLA are necessary for ketamine associated behavioral effects in the FST but not the NSF test.

### D1r agonist produces antidepressant responses

**Fig. 7:**
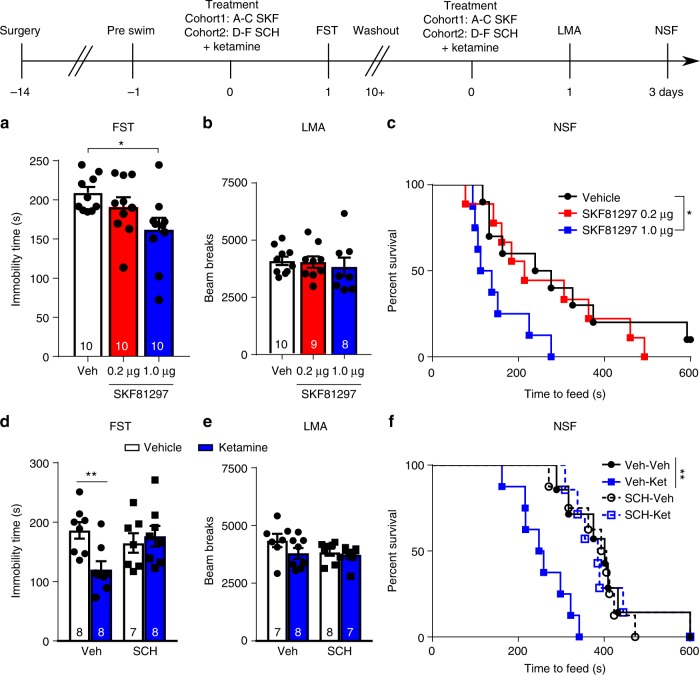
mPFC D1r pharmacological manipulations impact the effects of ketamine. **a** FST immobility time 24 h after D1r agonist administration (ANOVA *p* < 0.05, post-hoc *t*-test *p* *<* 0.05 vs. Veh). **b** Locomotor activity 24 h after D1r agonist infusion. **c** Time to feed in the NSF test 48 h after D1r agonist infusion (Mantel–Cox (log rank) overall *p* < 0.05, Veh vs. 1.0 µg *p* < 0.05). **d** FST immobility time 24 h after ketamine treatment with, or without, D1r antagonist (500 ng/side) pretreatment (Factorial ANOVA interaction *p* < 0.05, post-hoc *t*-test *p* < 0.01). **e** Locomotor activity 24 h after ketamine treatment with, or without, D1r antagonist pretreatment. **f** NSF time 72 h after treatment with combinations of ketamine and D1r antagonist (Mantel–Cox (log rank) overall *p* < 0.001, Veh–Veh vs. Veh–Ket *p* < 0.01); *p < 0.05, **p < 0.01. Error bars represent mean ± SEM. All data points—male

Our results suggest that *Drd1* cells are critical for a rapid antidepressant response, but it is unclear whether dopamine D1r signaling plays a role in the response to ketamine. Ketamine rapidly increases extracellular dopamine, as well as glutamate in the mPFC^3^, and D1r activity could contribute to the facilitation of AMPA currents required for the response to ketamine, as well as other rapid acting antidepressants^27,28^. Prior research has demonstrated that systemic administration of a D1r agonist produces antidepressant responses^29^, but it is unclear whether D1r agonist infusion into the mPFC produces ketamine like rapid behavioral actions. Here we found that infusion of a selective D1r agonist (SKF81297) into the mPFC decreased immobility time in the FST when tested 24 h after dosing (Fig. 7a). After a 13-day washout period, these same mice were administered a second infusion of SKF81297, which had no effect on locomotor activity tested 24 h after infusion (Fig. 7b), but significantly decreased the latency to feed in the NSF test 48 h later (Fig. 7c, home cage food consumption, Supplementary Fig. 7H).

In a second cohort of mice, the role of D1r in the actions of ketamine was also tested by pre-infusion (10 min) of a selective D1r antagonist into the mPFC (SCH39166, 500 ng per side). Infusion of SCH39166 completely blocked the antidepressant response to systemic ketamine (decreased immobility) in the FST determined 24 h after dosing (Fig. 7d); there was no effect on locomotor activity 24 h after systemic ketamine administration with or without SCH39166 treatment (Fig. 7e). Pre-infusion of SCH39166 also completely blocked the actions of ketamine in the NSF test, determined 72 h after dosing (Fig. 7f, home cage food consumption, Supplementary Fig. 7I).

## Discussion

Ketamine’s rapid antidepressant response includes a glutamate burst in the mPFC that is necessary for sustained antidepressant and anxiolytic outcomes. Previous work has demonstrated that photostimulation of pyramidal cells in the mPFC is sufficient to reproduce rapid antidepressant actions^4^ though the pyramidal cell subtype and output structures that mediate these effects have not been determined. Here we show that stimulation of *Drd1*-expressing cells in the mPFC produces rapid and sustained antidepressant responses observed up to 7 days after photostimulation. Additionally, our results demonstrate that *Drd1* cells, and activation of mPFC D1r, are necessary for the rapid antidepressant actions of ketamine. In contrast, stimulation of *Drd2*-expressing cells was neither sufficient nor necessary for the rapid antidepressant actions of ketamine. Finally, our results demonstrate that photostimulation of mPFC *Drd1* projections to the BLA recapitulate the effect of somatic stimulation, and that inhibition of these projections limits the response to ketamine. These findings demonstrate that a specific subpopulation of principal neurons, *Drd1* cells, acts as a driver of activity in a distributed circuit, capable of producing rapid and sustained antidepressant and anxiolytic behaviors.

**Fig. 8:**
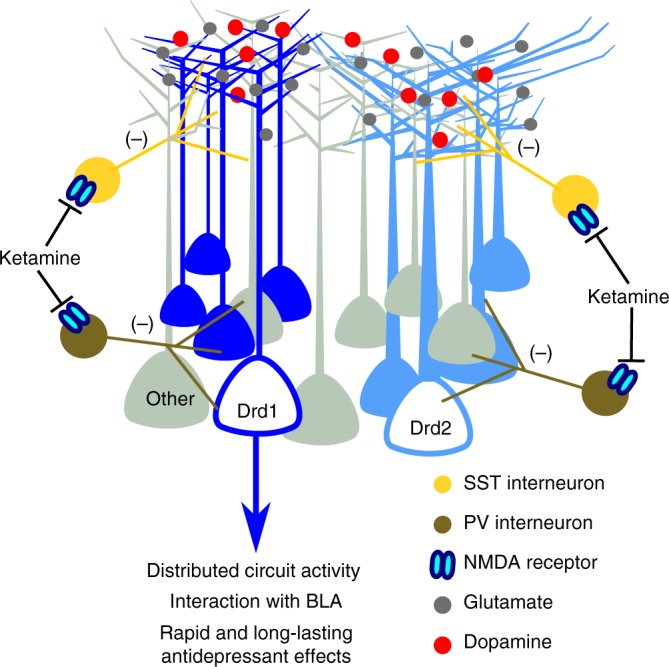
Proposed model of ketamine associated activity at *Drd1* cells in the mPFC. Antagonism of NMDA receptors on GABAergic interneurons produces a non-specific glutamate burst. Activation of *Drd1* expressing pyramidal cells modulates activity in output structures. mPFC *Drd1* cell projections to the BLA play a role in the antidepressant response to photostimulation and ketamine, but are likely not the only downstream targets of mPFC activation. *Drd1* cell activity concurrent with D1r activation results in rapid and long-lasting antidepressant effects. SST somatostatin, PV parvalbumin

Previous mechanistic studies have demonstrated that ketamine rapidly increases intracellular signaling in the mPFC that leads to morphological and physiological changes that persist well after ketamine metabolism and clearance. Sub-anesthetic ketamine doses produce an increase in mPFC glutamate that is followed by AMPA receptor activation, BDNF release, and increased mTORC1 signaling within 30–60 min (for review, see ref. ^30^). Following this initial response, there are increases in synaptic proteins including GluA1, spine density, and excitatory post synaptic currents, along with sustained antidepressant behavioral responses (up to 7 days)^31^. An increase in extracellular dopamine is also observed in the mPFC shortly after systemic ketamine administration^3^, though a role for dopamine in the rapid and sustained response to ketamine has not been investigated. Our pharmacological studies show the importance of D1r activation in the antidepressant effects of ketamine, a previously uninvestigated functional role of dopamine signaling. Increased D1r signaling in the mPFC could contribute to the synaptic actions of ketamine, including increased GluA1 levels^32^ as D1r activation augments glutamate-stimulated AMPA receptor insertion and facilitates AMPA activity^27,28^. These studies indicate that stimulation of glutamate-AMPA receptors on *Drd1-*expressing neurons is associated with stimulation of D1r signaling, which together are required for the rapid and sustained antidepressant actions of ketamine (Fig. 8). Also relevant is evidence that chronic stress exposure decreases dopamine levels in the mPFC^33^, and inhibition of mesolimbic dopamine transmission increases susceptibility in rodent social defeat models^15,34^. It will be interesting in future studies to determine if *Drd1* neurons and D1r signaling also play a key role in the actions of other rapid acting agents, including scopolamine and rapastinel, that act via glutamate and AMPA receptor activation^35,36^.

The integration of mPFC *Drd1*-expressing cells into larger brain networks appears critical for the induction of the rapid antidepressant response. Somatic stimulation of mPFC *Drd1* cells increased cFos expression in the BLA and extended amygdala (BNST), an effect that was absent in *Drd2-Cre* animals. Photostimulation of the mPFC *Drd1* terminal field in the BLA resulted in rapid and sustained antidepressant effects. The BLA has a well-documented role in fear expression, regulates emotional behaviors in real time^23,37^, and displays stress associated plasticity^38^. The BLA also has projections to and from other regions previously implicated in rapid antidepressant responses, including the ventral hippocampus^39,40^, BNST^41^, and mPFC^42^ and is well connected to impact behavioral responses to rapid acting agents. Notably, recent work suggests that mPFC projections onto BLA projection neurons preferentially target those connected to mPFC and ventral hippocampus^25^. It will be interesting to determine if these synapses are targeted by mPFC *Drd1* neurons, and how their function adapts following stimulation. For instance, recent work suggests that bursting activity in the lateral habenula, which receives limited projections from the mPFC^43^, can be regulated by ketamine administration to reduce depression-like behavior^43^. Further investigation is necessary to fully understand the circuit complexities underlying both the rapid and sustained antidepressant actions of ketamine. However, our results clearly demonstrate that current therapeutic options that impact circuit function such as transcranial magnetic stimulation and deep brain stimulation could benefit from technologies and approaches that allow greater regional specificity of stimulation.

Previous studies demonstrate a role for *Drd1* cells in the mPFC in temporal processing^44^, and their connections to BLA have been implicated in feeding^24^. The current study did not examine real time *Drd1* regulation of depression-like behaviors as locomotor activity was increased when the *Drd1* cells were stimulated in freely moving mice. In the behavioral paradigms utilized, particularly the FST, increased locomotor activity would be expected to decrease immobility, producing a false-positive antidepressant response. For this reason, the behavioral studies were conducted 24 h after stimulation when increased locomotor activity is no longer a confound. The sustained effects of photostimulation indicate that long-term neuroplasticity of *Drd1*-expressing neurons underlies the rapid and sustained antidepressant actions. This hypothesis is consistent with the rapid and sustained antidepressant actions of ketamine in depressed patients that are not observed until 2 h after dosing, when the drug has been largely metabolized, but are then sustained for up to 7 days^2^. Future studies utilizing approaches for monitoring cell type-specific activity could shed light on the real time regulation of behavior by *Drd1* and *Drd2* neurons.

It should be noted that the investigations reported do not include demonstrations of antidepressant responses in typical preclinical depression models that impact mPFC morphology and function such as chronic unpredictable stress^32^ or chronic restraint stress^45^. However, manipulations in the current paper occur in single housed animals that undergo surgical procedures followed by 2–4 weeks of single housing prior to behavioral testing. Single housing has been demonstrated to exacerbate anxiety and depression-like behavior in preclinical models^46–48^, and impact physiology in cortico-limbic circuits^49^. Additionally, the ketamine-associated glutamate burst appears to occur in healthy control subjects in clinical studies^50^ and in unstressed rodents in preclinical studies^51^. Therefore, while it will be important to study the impact of mPFC *Drd1-*expressing cells in chronic stress paradigms, the models used in the current study provide important information on the cell type and projection regions underlying rapid and sustained antidepressant responses.

In summary, the results demonstrate that driving neuronal activity in the *Drd1*-expressing subset of pyramidal cells in the mPFC is sufficient and necessary to induce the rapid and sustained antidepressant actions of ketamine. The mPFC acts as a node that coordinates the activity of downstream target regions that control emotion, anxiety, and mood, and the results indicate that the *Drd1* neurons within this node can stimulate plasticity that in turn produces rapid and sustained antidepressant actions in behavioral measures of despair and anxiety. The results also demonstrate a key role for the *Drd1* pyramidal cell population in the antidepressant actions of ketamine, which could lead to identification of new *Drd1* cell specific targets that have circuit level antidepressant impact, but without the dissociative and psychotomimetic side effects of ketamine. Toward this goal, gene expression profiling of *Drd1* expressing neurons is currently being conducted to identify receptors and channels that are expressed or enriched and that might be targeted for selective regulation of the *Drd1* pyramidal cell population.

## Methods

### Animals

Experimental subjects were 8–12-week-old males, and females, *Drd1-Cre* (Gensat EY262), *Drd2-Cre* (Gensat ER44) mice and WT littermates bred on a C57BL/6J background, or C57BL/6J mice (Jackson Laboratories, Bar Harbor ME). Mice were housed under 12 h light–dark cycle with ad libitum access to water and rodent chow. Animals were group housed until surgery. Animal use and procedures were in accordance with the National Institutes of Health Guide for the Care and Use of Laboratory Animals and approved by the Yale University Animal Care and Use Committees.

### Viral constructs

Adeno-associated viruses (AAV2-EF1a-DIO-hChR2(H134R)-EYFP, AAV2-EF1a-DIO-eARCH3.0-EYFP, AAV2-hSyn-EYFP) were obtained from the University of North Carolina vector core (~1–4 × 10^12^ vg/ml) or Addgene (AAV2-hSyn-DIO-hM4D(Gi)-mCherry, AAV2-hSyn-DIO-mCherry, AAVrg-PGK-Cre, ≥4 × 10^12^ vg/ml).

### Surgical procedures

For all stereotaxic surgeries, the animals were anesthetized with a ketamine/xylazine (120 mg/kg/10 mg/kg) cocktail. Animals were administered a subcutaneous injection of carprofen (5 mg/kg, Zoetis, Parsippany, NJ) prior to surgery, and received additional injections for 2 days following surgery. Once anaesthetized, fur at the incision site was removed and eyes were coated with ophthalmic ointment. Next, the animals were headfixed in a stereotaxic apparatus (David Kopf, Tujunga, CA) and the incision site was sterilized. A pair of craniotomies were made and a Hamilton syringe (Reno, NV) fitted with a 28 gauge needle was used to place a viral bolus (500 nl) at the following coordinates in millimeters within the mPFC (AP: 1.9, ML: 0.4, DV −2.7). Bilateral fiber optic cannula (0.7 mm center-to-center, 200 µM, 0.22 NA, Doric, Quebec) were implanted 0.2 mm above the viral bolus. Cannula were fixed to the skull using dental cement and a pair of skull screws. Following surgery, the animals were individually housed to prevent dislodging of cannula. Surgeries targeting the BLA followed the same procedure at the following coordinates; AP: −1.8, ML: 3.2, DV −4.8 for virus, −4.6 for fiber optic cannula. Drug infusion experiments followed the same procedure as above. Bilateral stainless-steel guide cannula (0.8 mm center-to-center, 26 gauge, Plastics One, Roanoke, VA) was placed within the mPFC (AP: 1.9, ML: 0.4, DV -2.5). Dummy cannula and a dust cap were employed to maintain guide-cannula patency.

### Behavior

Behavioral testing began 2–4 weeks after surgical procedures. Multiple tests meant to assess the potential for Cre-associated behavioral changes, and depression- and anxiety-like behaviors following experimental manipulations, were conducted along experimental timelines detailed in figures. All behaviors were conducted between 0900 and 1500 h. Animals were habituated to testing rooms for 30 min prior to experimentation. Animals were assigned to treatment based on genotype, and randomly assigned to drug treatments. Where a washout period was employed, treatments were counterbalanced to avoid confounding previous treatment.

For locomotor activity, the animals were placed into clean home cages (25 cm × 15 cm × 12 cm) for 30 min. Automated video tracking was employed to determine the activity levels (Anymaze, Stoelting, Wood Dale, IL). Where beam breaks are reported, the animals were placed into clean home cages and the activity was monitored by infrared beam break (Med Associates). For real time activity measurement during optogenetic stimulation and inhibition, the animals were placed into clean home cages and allowed to habituate for 20 min. Following the habituation period, a 10-min stimulation or inhibition period was initiated with alternating minute-long periods of light application as described below. A second optogenetic inhibition cohort was placed into clean home cages with light application (10 min) initiated immediately upon cage entry.

For forced swim, the animals were placed into 25 °C (±2 °C) water for 6 min. Immobility was scored by a blind observer and defined as a lack of activity except that necessary to keep the head above water. Water depth was such that the animals could not make contact with the bottom of the swim tank. We utilized a two-swim exposure paradigm to produce increased immobility during the test swim^52^. The full 6 min swim period was scored. Swim exposures were separated by 48 h. Antidepressant efficacy was also tested in photostimulated *Drd1-Cre* given a single 6-min swim 7 days after photostimulation.

For EPM, the animals were placed in the center of a plus-shaped maze (61 cm arm length, 6.5 cm arm width, 25 cm wall height, 45 cm above the floor) with a pair of open and closed arms and allowed to explore for 10 min. Light (300 lux) was directed evenly across the maze from above. Scoring was conducted in real time by a blind observer.

For NSF, the animals were food deprived for 20 h (16 h for D1 agonist experiment) prior to exposure to a novel arena (40 cm × 40 cm) with a small (2 g) chow pellet in the center. Time to feed was assessed in real time by a blind observer. Testing was followed by a 30-min home cage feeding test with ad lib access to pre-weighed chow to ensure that hunger was comparable across conditions.

For sucrose preference, the animals were habituated to 1% sucrose for 48 h prior to testing. Water was returned for 24 h on the third day. On the test day, the animals were provided with a two-bottle choice overnight and the preference for sucrose was calculated (sucrose preference = 100×((sucrose−water)/total). Bottle locations were counterbalanced, and the individual measuring intake was blind to experimental condition. Solutions were provided in ball valve sipper tubes to prevent leaking.

### Optogenetic manipulations

Optogenetic manipulations took place 24 h before the start of behavioral testing. The animals were coupled to a two-channel fiber optic patch cord (200 µM, 0.22 NA, Doric, Quebec) connected to a dual-channel fiber optic rotary joint (Doric, Quebec). The rotary joint was connected via the patch cord (200 µM, 0.22 NA, Doric, Quebec) to a 473-nm laser (Optoengine, Midvale, UT) for optogenetic stimulation. For photostimulation experiments, stimulation was conducted in the home cage for a 60-min period (1 min on/1 min off) with 15 ms pulses of 473 nm light (5 mW/side, reduced if uncoordinated behavior was observed) at 10 hz during the on periods. The 10 hz pulse rate was chosen based on previous work demonstrating high spike fidelity at this rate^18^. For cFos experiments, the above stimulation paradigm was used for a 20-min period 60 min before perfusion (4% PFA). For photoinhibition experiments, constant 561 nm (5 mW/side, Optoengine, Midvale, UT) light was directed at the mPFC. Others have demonstrated that 30 min of constant light at 20 mW does not cause tissue injury, and have used up to 90 min of constant light at 4–8 mW for inhibition experiments^53^. To capture the active period following ketamine administration, the laser was activated immediately following ketamine administration and remained on for 60 min.

### mPFC drug infusion

Drug infusion took place 24 h prior to the start of behavioral testing. Infusion cannula (28 gauge, Plastics One, Roanoke, VA) were cut to extend 0.2 mm beyond the implanted guide-cannula targeting the mPFC. A bolus of drug or vehicle (0.5 µL per side) was administered over a 5-min injection period using a syringe pump (Harvard Apparatus, Holliston, MA). The cannula were left in place for 5 min before removal to allow for diffusion of drug. Systemic ketamine treatment took place 10 min after infusion in D1 antagonist experiments.

### Pharmacological agents

Ketamine (Tocris, Bristol, UK) was administered intraperitoneally at 10 mg/kg in a 10 ml/kg volume. Intraperitoneal clozapine-*n*-oxide (Enzo Scientific, NY, USA) was administered at 1 mg/kg in a 10 ml/kg volume. The D1 agonist SKF81297 (Tocris, 0.2 µg per side or 1.0 µg per side) and D1 antagonist SCH39166 (Tocris, 500 ng per side) were infused directly into the mPFC. All compounds were diluted in 0.9% saline.

### Immunohistochemistry

The animals were perfused with PBS followed by 4% paraformaldehyde. Following 48 h of post-fixation sucrose (25% sucrose in PBS), the tissue was sectioned at 20 µM. Following PBS washes and 3 h block (PBS, 2% triton, 3%NGS), the sections were incubated overnight with primary antibodies (cFos: SC-52, 1:1000, Santa Cruz Biotechnology, USA; NeuN; MAB377, 1:1000, MilliporeSigma, USA). PBS washes were followed by 2-h secondary antibody incubation (Alexa Fluor conjugated goat-anti-mouse and goat-anti-rabbit, Invitrogen, Carlsbad, CA) followed by three additional washes. Finally, the tissue was mounted with Vectashield mounting medium (Vector Laboratories, Burlingame, CA). Samples were imaged on a Zeiss Axioskop2 (Zeiss, Germany) at 10x using a FITC/TRITC filter set. Images were obtained using Zeiss Zen2 software. Images were processed to remove the background in ImageJ (NIH). For unilateral stimulation, each image included both stimulated and unstimulated hemisphere and background subtraction was applied to the entire image. For cFos quantification, the average cell count from 2–3 sections per animal was obtained.

### Electrophysiology

Whole-cell recordings from mPFC pyramidal cells in 400 µM brain slices of mice previously injected with AAV2-EF1a-DIO-hChR2(H134R)-EYFP or AAV2-EF1a-DIO-eARCH3.0-EYFP were obtained. Photostimulation/inhibition was conducted as described above. Postsynaptic currents were recorded in voltage clamp, clamped near resting potential (~75 mV).

### Statistical analysis

The statistical tests used and the outcomes are presented in the figure legends. All statistical tests were conducted in a two-tailed fashion in Graphpad version 7 (La Jolla, CA). Sample sizes were chosen based on previous experience with the behavioral tests employed, and power analysis (G*Power, Universitat Dusseldorf) conducted following a pilot study (not reported). In all cases, sample sizes meet or exceed suggested sample size based on the power analysis. Due to low sample size, sex difference analysis was not conducted in experiments containing female mice. Three mice (two *Drd1-Cre*, one *Drd1-WT*) were removed from analysis for failure to display any immobility during initial forced swim exposure. One mouse was removed from NSF results in the D1 agonist experiment (1.0 µg) due to feeding time >2 SD from the group mean. Where appropriate, non-parametric statistics are employed. The details for all statistical tests are found in Supplementary Table 1.

### Reporting Summary

Further information on experimental design is available in the Nature Research Reporting Summary linked to this article.

## Supplementary information

Supplementary Information

Reporting Summary

## Data Availability

The data corresponding to the studies detailed here are available from the lead contact upon reasonable request.
