# Supplementary material for: Optogenetic stimulation of medial prefrontal cortex Drd1 neurons produces rapid and long-lasting antidepressant effects
Source: Nat Commun. 2019 Jan 15;10:223. doi: 10.1038/s41467-018-08168-9 (PMC6333924; doi:10.1038/s41467-018-08168-9)
Supplement: Supplementary file 1 — Supplementary Information [file 41467_2018_8168_MOESM1_ESM.pdf]

Supplementary Information

Optogenetic stimulation of medial prefrontal cortex *Drd1* neurons produces rapid and long-lasting antidepressant effects

Hare et al.

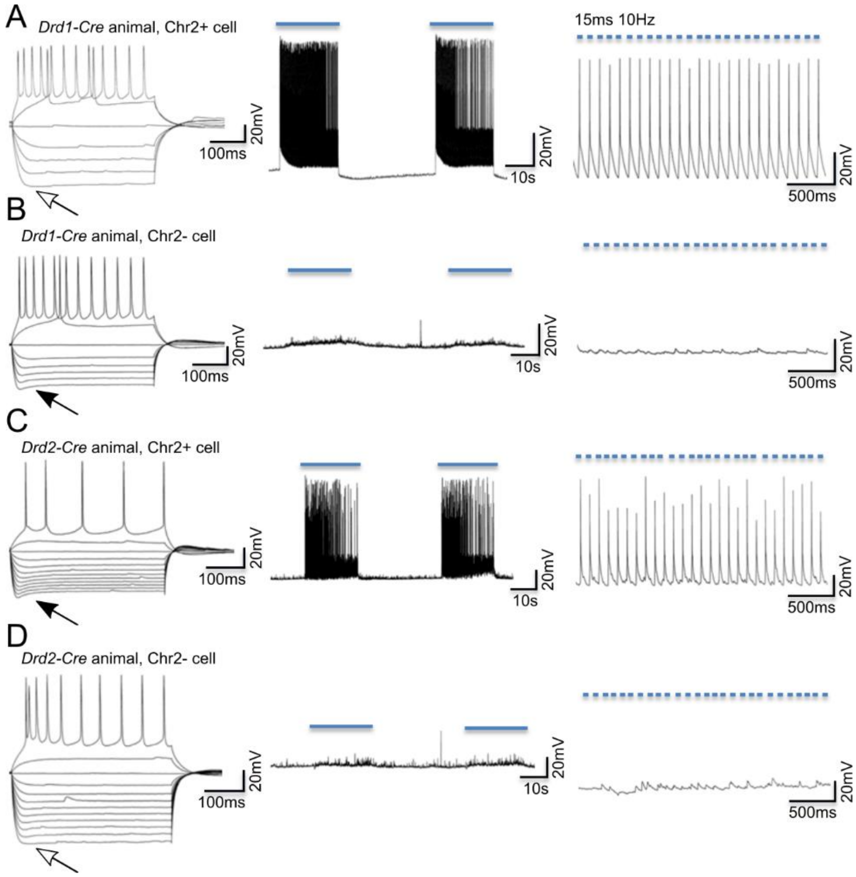

Supplementary Figure 1: Recordings from *Drd1-Cre* and *Drd2-Cre* mice suggest separable populations. (A,B) Light sensitive cells in *Drd1-Cre* mice demonstrated low voltage sag (open arrow) in response to hyperpolarizing current injections in comparison to cells that did not generate action potentials in response to 473nm light application (putative D2/typeA, dark arrow).
(C,D) In *Drd2-Cre* mice light sensitive larger voltage sag cells were found along with light insensitive reduced voltage sag cells (putative D1/typeB). Representative traces from male mice.

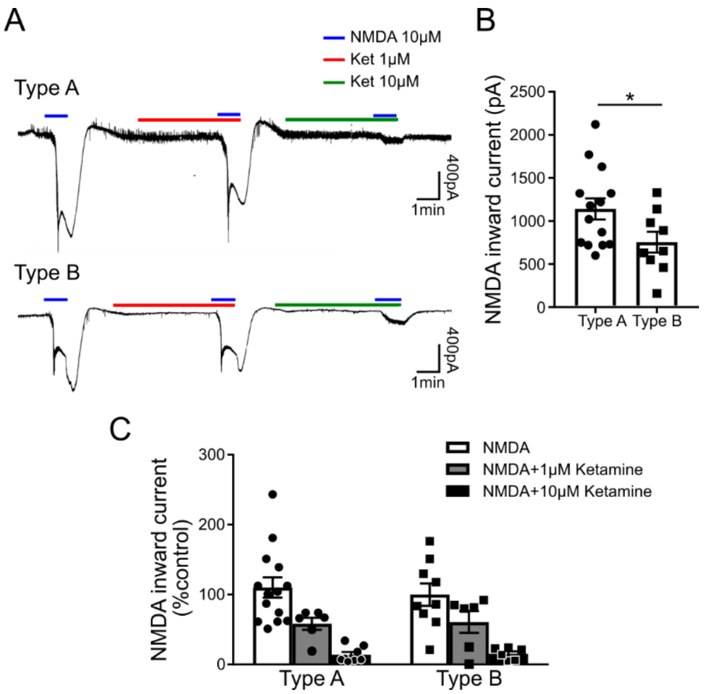

Supplementary Figure 2: Ketamine regulates NMDA associated currents in type A and type B cells.
(A) Example traces of NMDA (10 µM) associated inward current prior to, and during, bath application of different concentrations of ketamine.
(B) NMDA associated inward current magnitude in type A and type B cells (t-test, $p<0.05$).
(C) Blockade of inward current following ketamine application in type A and type B cells (factorial ANOVA, ketamine incubation $p<0.0001$). Type A – 3 animals, Type B - 3 animals, all male. Data points represent individual cells. Error bars represent mean±SEM, *$p<0.05$

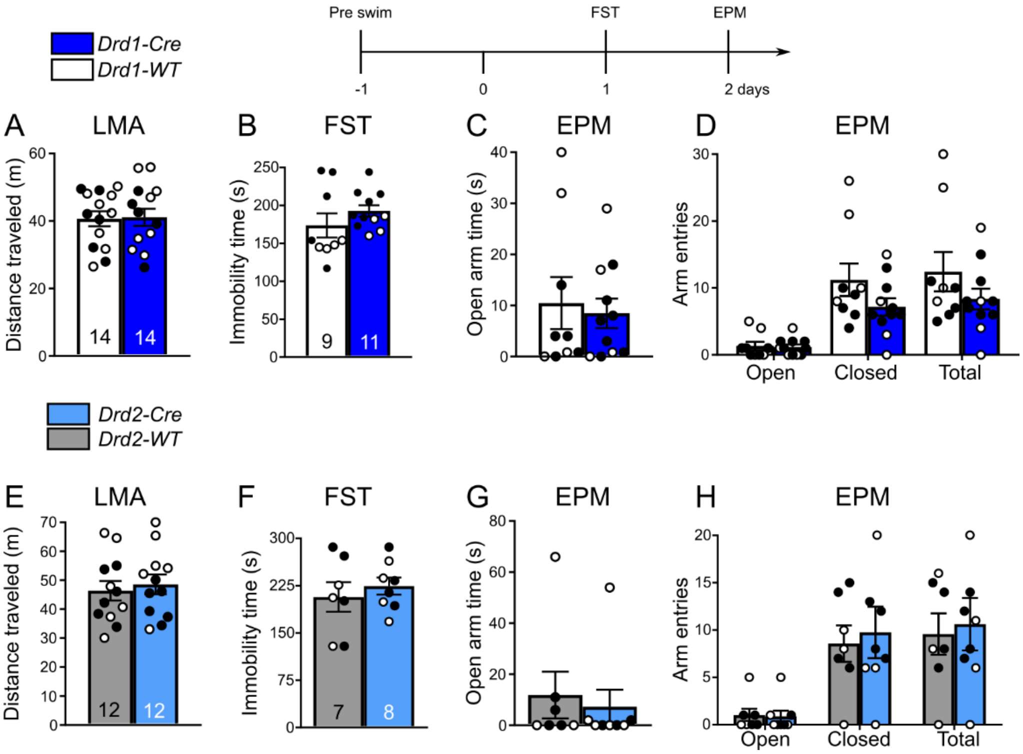

Supplementary Figure 3: Cre-associated endophenotype is absent in *Drd1-Cre* and *Drd2-Cre* mice.
(A,E) Locomotor activity recorded for 20 minutes in a novel home cage.
(B,F) Forced swim immobility time during a six minute test swim.
(C,G;D,H) elevated plus maze open arm time and plus maze activity.
Drd1-Cre n= (A) 8 females, 6 males per genotype, (B-D) 7 male, 4 female; WT 5 male, 4 female.
Drd2-Cre n= (E) 6 females, 6 males per genotype, (G-J) 4 male, 4 female; WT 4 male, 3 female. Error bars represent mean ± SEM. Male – closed circles, Female – open circles

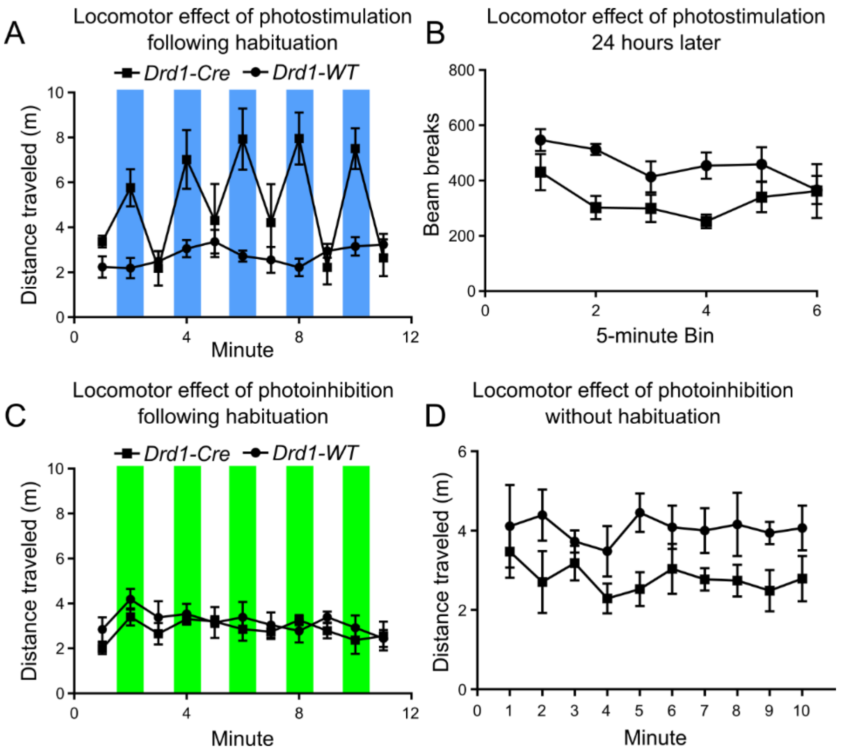

Supplementary Figure 4: Locomoion during photostimulation and photoinhibition of *Drd1-Cre* mice.
(A) Photostimulation associated increases in locomotor activity in animals habituated to a novel cage for 10 minutes (Factorial ANOVA, $p<0.0001$, n=7/group, all male).
(B) Locomotor activity in animals prior to and following photostimulation as employed in behavioral experiments (Factorial ANOVA, interaction $p<0.05$, n=5/group, all male).
(C) Photoinhibition associated locomotor activity in animals following a 10 minute habituation period (n=8/group, all male).
(D) Locomotor activity during photoinhbition initiated immediately upon entry to a novel cage (Factorial ANOVA, group p<0.05, n=8 *Drd1-Cre*, 6 male, 2 female, 6 *Drd1-WT,* 4 male, 2 female), *p<0.05. Error bars represent mean ± SEM.

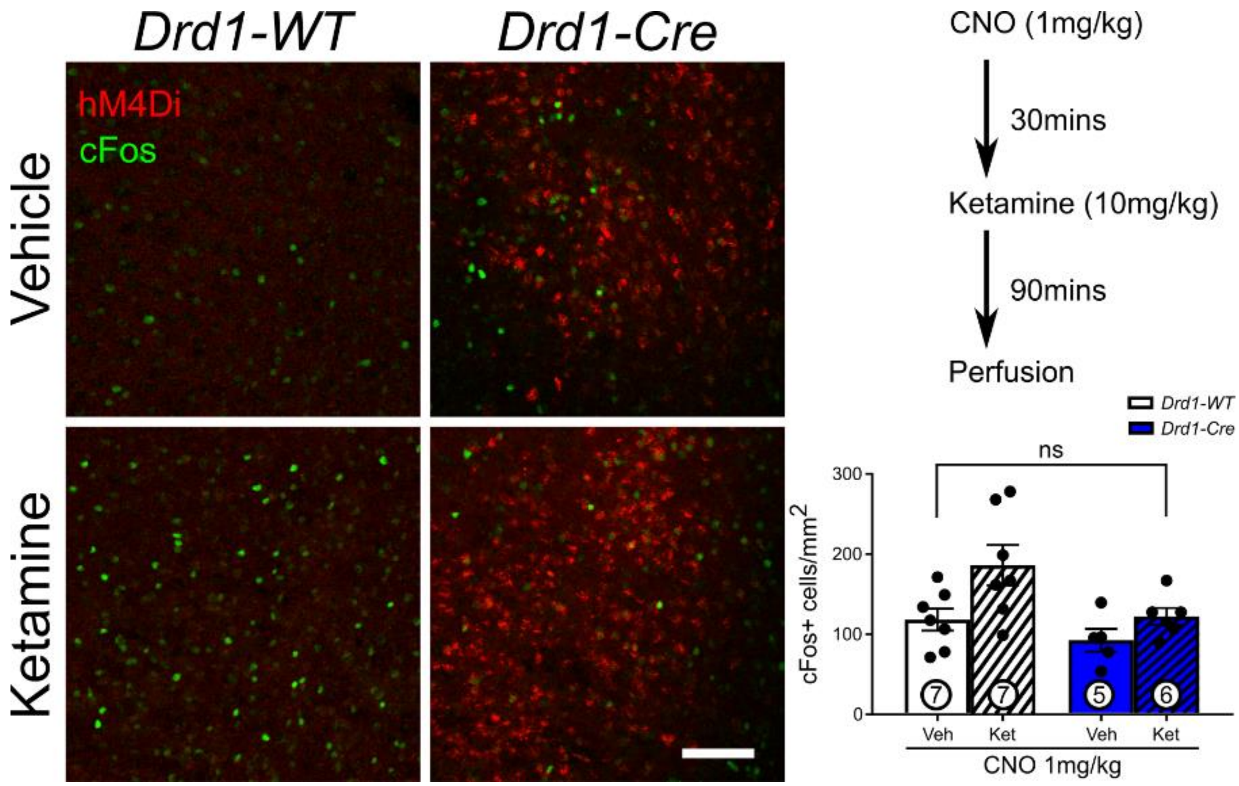

Supplementary Figure 5: Inhibition of mPFC *Drd1* expressing cells blocks ketamine induction of cFos immunopositive neurons (normalized cells per mm^2) in the mPFC. Factorial ANOVA genotype $p<0.05$, treatment $p<0.05$. All data points male. Error bars represent mean ± SEM, scale bar 100uM, red = hM4Di, green = cFos.

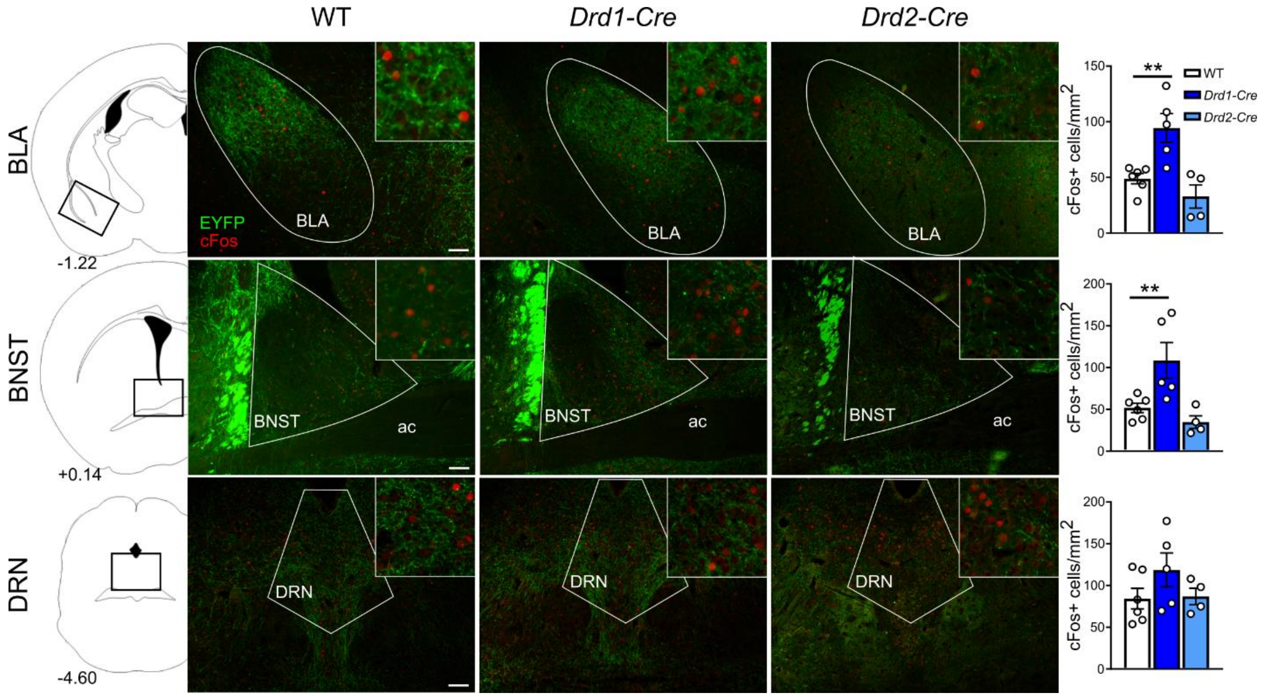

Supplementary Figure 6: Photostimulation of mPFC *Drd1* and *Drd2* expressing cells differentially stimulate cFos labeling (normalized cells per mm^2) in target regions. BLA, basolateral amygdala, BNST, bed nucleus of the stria terminalis, DRN, dorsal raphe nucleus, AC, anterior commisure. **=$p<0.01$, error bars represent mean ± SEM, scale bar 100uM. All data points male.

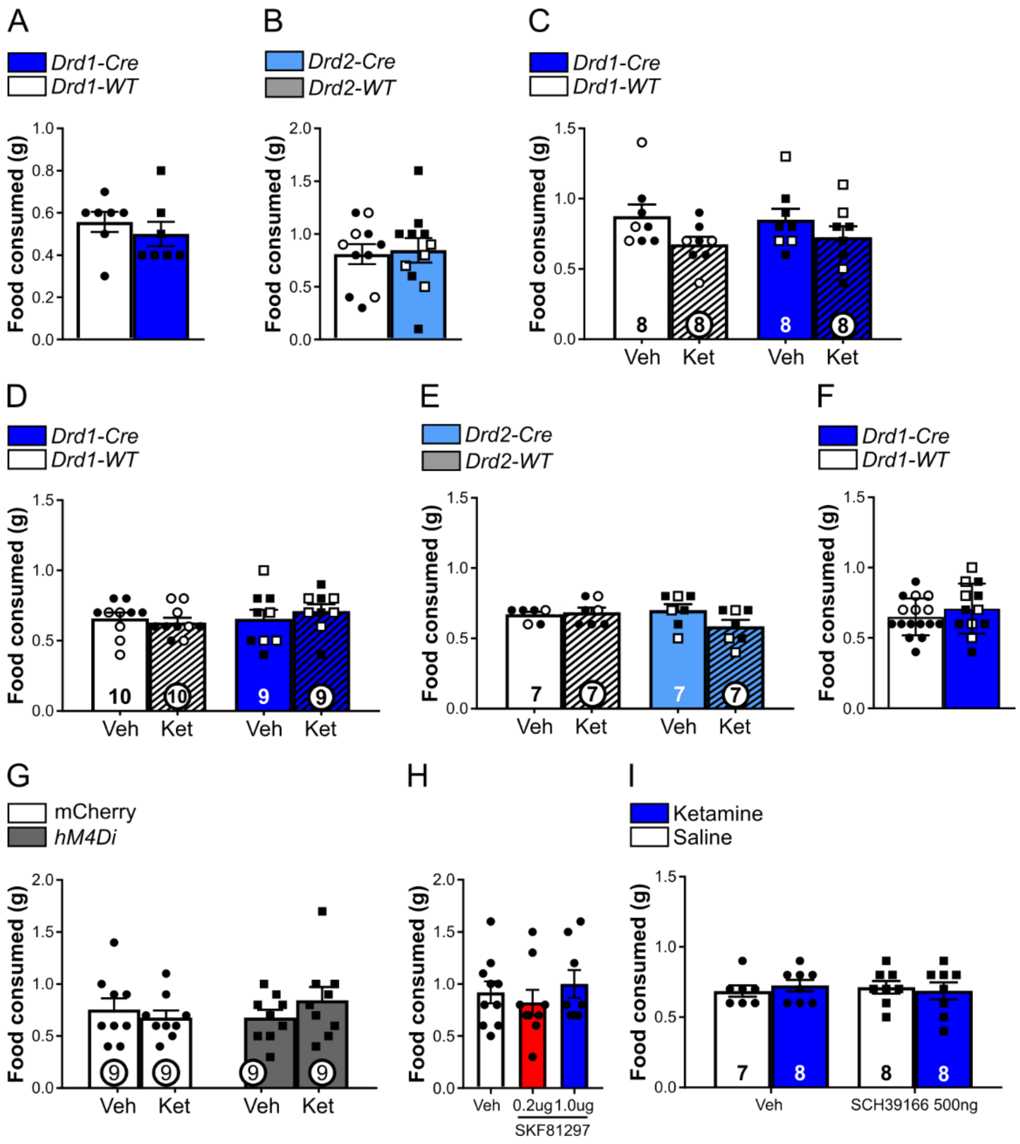

Supplementary Figure 7: Home cage feeding results following novelty suppressed feeding (NSF) tests.
(A) Home cage feeding following NSF test depicted in Figure 2F
(B) Home cage feeding following NSF test depicted in Figure 2L
(C) Home cage feeding following NSF test depicted in Figure 3F
(D) Home cage feeding following NSF test depicted in Figure 3I
(E) Home cage feeding following NSF test depicted in Figure 4C
(F) Home cage feeding following NSF test depicted in Figure 5D
(G) Home cage feeding following NSF test depicted in Figure 6C
(H) Home cage feeding following NSF test depicted in Figure 7C
(I) Home cage feeding following NSF test depicted in Figure 7F
Statistical results for these comparisons are found in Supplementary Table 1 with the relevant NSF results. Error bars represent mean ± SEM. Data points represent individual animals. Male – closed data points, Female – open data points

Supplementary Table 1: Statistical results

Figure	test	Comparison	Statistic	p-value
1D	Wilcoxon matched pairs signed rank	*Drd1*stim+V *Drd1*stim-	*W=21, n=6*	*p*<0.05
		*Drd2*stim+V *Drd2*stim-	*W=21, n=6*	*p*<0.05
	Mann Whitney	*Drd1*Stim+ V WT	*U=0.0*	*p*<0.001
	Mann Whitney	*Drd2*Stim+ V WT	*U=0.0*	*p*<0.001
1E	2way ANOVA (genotypeXcurrent)	genotype	*f*(7,116)=47.41	*p*<0.0001
		current	*f* (7,116)=11.93	*p*<0.0001
		interaction	*f* (7,116)=0.32	*p*=0.94
S2B	t-test	typeA V typeB	*t* (21)=2.18	*p*<0.05
S2C	2way ANOVA (genotypeXcurrent)	treatment	*f*(2,44)=21.52	*p*<0.0001
		cell type	*f* (1,44)=0.01	*p*=0.91
		interaction	*f* (2,44)=0.004	*p*=0.99
S3A	t-test	*Drd1*Cre V *Drd1WT*	*t* (26)=1.20	*p*=0.91
S3B	t-test	*Drd1*Cre V *Drd1WT*	*t* (18)=1.15	*p*=0.27
S3C	t-test	*Drd1*Cre V *Drd1WT*	*t* (18)=0.39	*p*=0.70
S3D	t-test (EPM open entries)	*Drd1*Cre V *Drd1WT*	*t* (18)=0.22	*p*=0.83
	t-test (EPM closed entries)	*Drd1*Cre V *Drd1WT*	*t* (18)=1.54	*p*=0.14
	t-test (EPM total entries)	*Drd1*Cre V *Drd1WT*	*t* (18)=1.30	*p*=0.24
S3E	t-test	*Drd2*Cre V *Drd2*WT	*t* (22)=0.46	*p*=0.65
S3F	t-test	*Drd2*Cre V *Drd2*WT	*t* (13)=0.66	*p*=0.52
S3G	t-test	*Drd2*Cre V *Drd2*WT	*t* (13)=0.41	*p*=0.69
S3H	t-test (EPM open entries)	*Drd2*Cre V *Drd2*WT	*t* (13)=0.14	*p*=0.89
	t-test (EPM closed entries)	*Drd2*Cre V *Drd2*WT	*t* (13)=0.74	*p*=0.74
	t-test (EPM total entries)	*Drd2*Cre V *Drd2*WT	*t* (13)=0.29	*p*=0.78
2A	t-test	*Drd1*Cre V *Drd1WT*	*t* (18)=2.48	*p*<0.05
2B	t-test	*Drd1*Cre V *Drd1WT*	*t* (18)=2.59	*p*<0.05
2C	t-test (EPM open entries)	*Drd1*Cre V *Drd1WT*	*t* (18)=1.96	*p*=0.07
	t-test (EPM closed entries)	*Drd1*Cre V *Drd1WT*	*t* (18)=0.68	*p*=0.50
	t-test (EPM total entries)	*Drd1*Cre V *Drd1WT*	*t* (18)=0.15	*p*=0.89
2E	t-test	*Drd1*Cre V *Drd1WT*	*t* (12)=0.01	*p*=0.99
2F	Mantel-Cox (log rank)	*Drd1*Cre V *Drd1WT*	X^2(1,N=14)=6.31	*p*<0.05
	t-test (home cage feeding)	*Drd1*Cre V *Drd1WT*	*t* (12)=0.76	*p*=0.46
2G	t-test	*Drd1*Cre V *Drd1WT*	*t* (12)=2.84	*p*<0.05
2I	t-test	*Drd2*Cre V *Drd2*WT	*t* (20)=0.09	*p*=0.93
2J	t-test	*Drd2*Cre V *Drd2*WT	*t* (20)=1.05	*p*=0.31
2K	t-test (EPM open entries)	*Drd2*Cre V *Drd2*WT	*t* (20)=2.35	*p*<0.05
	t-test (EPM closed entries)	*Drd2*Cre V *Drd2*WT	*t* (20)=1.47	*p*=0.16
	t-test (EPM total entries)	*Drd2*Cre V *Drd2*WT	*t* (20)=2.12	*p*<0.05
2L	Mantel-Cox (log rank)	*Drd2*Cre V *Drd2*WT	X^2(1,N=22)=0.05	*p*=0.82
	t-test (home cage feeding)	*Drd2*Cre V *Drd2*WT	*t* (20)=0.24	*p*=0.81
3E	2way ANOVA (genotypeXtreatment)	genotype	*f*(1,28)=0.38	*p*=0.54
		treatment	*f* (1,28)=5.04	*p*<0.05
		interaction	*f* (1,28)=2.64	*p*=0.11
3F	Mantel-Cox (log rank)	Overall	X^2(3,N=32)=14.91	*p*<0.01
	Mantel-Cox (log rank)	*Drd1*WT-Veh V *Drd1*WT-Ket	X^2(1,N=16)=4.91	*p*<0.05

	Mantel-Cox (log rank)	*Drd1*Cre-Veh V *Drd1*Cre-Ket	X^2(1,N=16)=1.56	*p=0.21*
	2way ANOVA (home cage feeding, genotypeXtreatment)	Genotype	*f*(1,28)=0.05	*p*=0.82
		treatment	*f* (1,28)=2.55	*p*=0.12
		interaction	*f* (1,28)=0.70	*p*=0.41
3H	2way ANOVA (genotypeXtreatment)	genotype	*f*(1,34)=2.84	*p*=0.10
		treatment	*f* (1,34)=4.94	*p*<0.05
		interaction	*f* (1,34)=13.20	*p*<0.001
	t-test (Bonferroni-posthoc)	*Drd1*WT-Veh V *Drd1*WT-Ket	*t* (18)=4.25	*p*<0.001
	t-test (Bonferroni-posthoc)	*Drd1*Cre-Veh V *Drd1*Cre-Ket	*t* (16)=0.97	*p*=0.68
3I	Mantel-Cox (log rank)	Overall	X^2(3,N=38)=9.88	*p*<0.05
	Mantel-Cox (log rank)	*Drd1*WT-Veh V *Drd1*WT-Ket	X^2(1,N=20)=7.65	*p*<0.01
	Mantel-Cox (log rank)	*Drd1*Cre-Veh V *Drd1*Cre-Ket	X^2(1,N=18)=0.59	*p*=0.44
	2way ANOVA (home cage feeding, genotypeXtreatment)	genotype	*f*(1,34)=0.66	*p*=0.42
		treatment	*f* (1,34)=0.07	*p*=0.78
		interaction	*f* (1,34)=0.82	*p*=0.37
S4A	2way ANOVA (genotypeXtime)	genotype	*f*(1,12)=17.14	*p*<0.01
		time	*f* (10,120)=4.20	*p*<0.0001
		interaction	*f* (10,120)=4.46	*p*<0.0001
S4B	2way ANOVA (genotypeXtime)	genotype	*f*(1,8)=4.03	*p=0.08*
		time	*f* (5,40)=4.70	*p*<0.01
		interaction	*f* (3,24)=2.49	*p*<0.05
S4C	2way ANOVA (genotypeXtime)	genotype	*f*(1,14)=0.60	*p=0.45*
		time	*f* (10,140)=2.28	*p*<0.05
		interaction	*f* (10,140)=0.64	*p=0.78*
S4D	2way ANOVA (genotypeXtime)	genotype	*f*(1,12)=5.16	*p<0.05*
		time	*f* (9,108)=0.56	*p*=0.83
		interaction	*f* (9,108)=0.45	*p=0.91*
S5	2way ANOVA (genotypeXtreatment)	genotype	*f*(1,21)=6.13	*p*<0.05
		treatment	*f* (1,21)=7.27	*p*<0.05
		interaction	*f* (1,21)=0.30	*p*=0.30
	t-test (Bonferroni-posthoc)	*Drd1*WT veh V *Drd1*Cre ket	*t* (11)=0.16	*p*>0.99
4B	2way ANOVA (genotypeXtreatment)	genotype	*f*(1,24)=2.18	*p*=0.15
		treatment	*f* (1,24)=24.64	*p*<0.0001
		interaction	*f* (1,24)=2.78	*p*=0.11
	t-test (Bonferroni-posthoc)	*Drd2*WT-Veh V *Drd2*WT-Ket	*t* (12)=2.33	*p*=0.057
	t-test (Bonferroni-posthoc)	*Drd2*Cre-Veh V *Drd2*Cre-Ket	*t* (12)=4.69	*P*<0.001
4C	Mantel-Cox (log rank)	Overall	X^2(3,N=28)=22.30	*p*<0.0001
	Mantel-Cox (log rank)	*Drd2*WT-Veh V *Drd2*WT-Ket	X^2(1,N=14)=14.51	*p*<0.0001
	Mantel-Cox (log rank)	*Drd2*Cre-Veh V *Drd2*Cre-Ket	X^2(1,N=14)=6.41	*p<0.05*

	2way ANOVA (home cage feeding, genotypeXtreatment)	Genotype	*f*(1,24)=0.92	*p*=0.34
		treatment	*f* (1,24)=1.82	*p*=0.19
		interaction	*f* (1,24)=3.00	*p*=0.10
S6BLA	Kruskal-Wallis test	Overall	*H*(3)=10.10	*p*<0.001
	Mann Whitney	*Drd1* V WT	*U=0.5*	*p*<0.01
	Mann Whitney	*Drd2* V WT	*U=5*	*p=0.17*
S6BNST	Kruskal-Wallis test	Overall	*H*(3)=10.11	*p*<0.001
	Mann Whitney	*Drd1* V WT	*U=1*	*p*<0.01
	Mann Whitney	*Drd2* V WT	*U=4*	*p=0.11*
S6DRN	Kruskal-Wallis test	Overall	*H*(3)=2.57	*p*=0.29
	Mann Whitney	*Drd1* V WT	*U=7*	*P*=0.18
	Mann Whitney	*Drd2* V WT	*U=11*	*p=0.91*
5B	Mann Whitney	*Drd1*Cre V *Drd1*WT	*U=0*	*p*<0.01
5C	Mann Whitney	*Drd1*Cre V *Drd1*WT	*U=55.5*	*p*<0.05
5D	Mantel-Cox (log rank)	*Drd1*Cre V *Drd1*WT	X^2(1,N=28)=7.61	*p*<0.01
	t-test (home cage feeding)	*Drd1*Cre V *Drd1*WT	*t* (26)=1.00	*p*=0.33
5	NSF 72 hours	*Drd1*Cre V *Drd1*WT	X^2(1,N=28)=1.18	*p*=0.29
	Home cage feeding	*Drd1*Cre V *Drd1*WT	*t* (26)=0.38	*p*=0.71
6B	2way ANOVA (genotypeXtreatment)	group	*f*(1,33)=3.85	*p*=0.06
		treatment	*f* (1,33)=2.55	*p*=0.12
		interaction	*f* (1,33)=4.84	*p*<0.05
6C	Mantel-Cox (log rank)	Overall	X^2(3,N=36)=7.30	*p*=0.06
	Mantel-Cox (log rank)	mCherry-Veh V mCherry -Ket	X^2(1,N=18)=3.66	*p*=0.06
	Mantel-Cox (log rank)	hM4Di-Veh V hM4Di-Ket	X^2(1,N=18)=3.30	*p*=0.07
	2way ANOVA (home cage feeding, genotypeXtreatment)	group	*f*(1,32)=0.21	*p*=0.65
		treatment	*f* (1,32)=0.21	*p*=0.65
		interaction	*f* (1,32)=1.55	*p*=0.22
7A	ANOVA (SKF81297)	Veh,0.2ug, 1.0ug	*f* (2,27)=3.91	*p*<0.05
	t-test (Bonferroni-posthoc)	Veh V 1.0ug	*t* (18)=2.77	*p*<0.05
	t-test (Bonferroni-posthoc)	Veh V 0.2ug	*t* (18)=1.05	*p*=0.60
7B	ANOVA	Veh,0.2ug, 1.0ug	*f* (2,25)=0.15	*p=0.85*
7C	Mantel-Cox (log rank)	Overall	X^2(2,N=27)=7.77	*p*<0.05
	Mantel-Cox (log rank)	Veh V 1.0ug	X^2(1,N=18)=5.73	*p*<0.05
	Mantel-Cox (log rank)	Veh V 0.2ug	X^2(1,N=19)=0.34	*p=0.56*
	ANOVA(home cage feeding)	Veh,0.2ug, 1.0ug	*f* (2,24)=0.53	*p=0.59*
7D	2way ANOVA (KetamineXSCH39166)	Ketamine	*f*(1,27)=3.12	*p*=0.09
		SCH39166	*f* (1,27)=1.26	*p*=0.27
		interaction	*f* (1,27)=6.28	*p*<0.05
	t-test (Bonferroni-posthoc)	Veh/Veh Vs Veh/Ketamine	*t* (14)=3.08	*p*<0.01
	t-test (Bonferroni-posthoc)	Veh/Veh Vs SCH39166/Ketamine	*t* (14)=0.47	*p*=0.66
7E	2way ANOVA (KetamineXSCH39166)	Ketamine	*f*(1,26)=1.81	*p*=0.19
		SCH39166	*f* (1,26)=2.44	*p*=0.13
		interaction	*f* (1,26)=1.05	*p=0.32*
7F	Mantel-Cox (log rank)	Overall	X^2(3,N=30)=22.99	*p*<0.001

	Mantel-Cox (log rank)	Veh/Veh Vs Veh/Ketamine	$X^2(1,N=15)=8.82$	$p<0.01$
	Mantel-Cox (log rank)	Veh/Veh Vs SCH39166/Ketamine	$X^2(1,N=14)=0.01$	$p=0.93$
	2way ANOVA Home cage feeding (KetamineXSCH39166)	Ketamine	$f(1,27)=0.02$	$p=0.88$
		SCH39166	$f\ (1,27)=0.01$	$p=0.91$
		interaction	$f\ (1,27)=0.45$	$p=0.51$
